# Draft Genome Sequence of *Burkholderia* sp. Strain CCA53, Isolated from Leaf Soil

**DOI:** 10.1128/genomeA.00630-16

**Published:** 2016-07-07

**Authors:** Hironaga Akita, Zen-ichiro Kimura, Mohd Zulkhairi Mohd Yusoff, Nobutaka Nakashima, Tamotsu Hoshino

**Affiliations:** a Research Institute for Sustainable Chemistry, National Institute of Advanced Industrial Sciences and Technology (AIST), Kagamiyama, Higashi-Hiroshima, Hiroshima, Japan; b Department of Civil and Environmental Engineering, National Institute of Technology, Kure College, Kure, Hiroshima, Japan; c Department of Bioprocess Technology, Faculty of Biotechnology and Biomolecular Sciences, Universiti Putra Malaysia, Serdang, Selangor, Malaysia; d Bioproduction Research Institute, National Institute of Advanced Industrial Sciences and Technology (AIST), Tsukisamu-Higashi, Toyohira-ku, Sapporo, Japan; e Department of Biological Information, Graduate School of Bioscience and Biotechnology, Tokyo Institute of Technology, Ookayama, Meguro-ku, Tokyo, Japan

## Abstract

*Burkholderia* sp. strain CCA53 was isolated from leaf soil collected in Higashi-Hiroshima City in Hiroshima Prefecture, Japan. Here, we present a draft genome sequence of this strain, which consists of a total of 4 contigs containing 6,647,893 bp, with a G+C content of 67.0% and comprising 9,329 predicted coding sequences.

## GENOME ANNOUNCEMENT

The genus *Burkholderia* contains Gram-negative, non-spore-forming β-proteobacteria (1). This genus was separated from the former *Pseudomonas* rRNA homology group II, and more than 80 *Burkholderia* species have been reported to date (1). Based on phylogenetic analyses of the sequences of their 16S rRNA, *acdS*, *gyrB*, *recA*, and *rpoB* genes, as well as their genome sequences, *Burkholderia* species have been classified into two major clusters and several subgroups (2). Group A comprises plant-associated and saprophytic species, while group B contains opportunistic pathogens that infect animals, humans, and plants (2). *Burkholderia* sp. strain CCA53 was recently isolated from leaf soil and classified into group B (3). An important limitation of industrial host microorganisms, such as *Escherichia coli* and *Saccharomyces cerevisiae*, is an inability to assimilate lignin as a carbon source, but *Burkholderia* sp. CCA53 has this ability (3). Because of that, it is anticipated that *Burkholderia* sp. CCA53 could be a useful strain for industrial production of second-generation biofuels (3), as lignin is a widely distributed raw material on Earth (4). To enable gene engineering of *Burkholderia* sp. CCA53 for industrial applications, we determined its draft genome sequence.

A sample was prepared for sequencing by growing *Burkholderia* sp. CCA53 aerobically overnight at 37°C in Nutrient broth (Kyokuto). The genomic DNA was then extracted and purified using an illustra bacteria genomicPrep mini spin kit (GE Healthcare), according to the manufacturer’s instructions. The purity and concentration of the genomic DNA were measured using NanoDrop (Thermo Scientific) and a Quant-iT double-stranded DNA (dsDNA) BR Assay kit (Invitrogen). After fragmenting the genomic DNA (8 µg) into approximately 20-kb pieces using g-TUBE (Covaris), the resultant fragments were ligated to SMRTbell sequencing adapters using an SMRTbell Template Prep Kit 1.0 (Pacific Biosciences), yielding the SMRTbell libraries. The library size was measured using Agilent 2200 TapeStation (Agilent Technologies). The SMRTbell libraries were then bound to polymerases and sequencing primers using a DNA/Polymerase Binding Kit P6 version 2 (Pacific Biosciences), yielding the sequencing template. The concentration of the sequencing templates was calculated using Binding Calculator version 2.3.1.1 (Pacific Biosciences), after which the templates were bound to MagBeads using a MagBead Kit (Pacific Biosciences) and loaded onto single-molecule real-time (SMRT) Cell 8 Pac V3 (Pacific Biosciences). The sequencing was performed using PacBio RS II (Pacific Biosciences). The raw data were 65,150 reads at 138-fold coverage and were assembled *de novo* using SMRT Analysis version 2.3.0 (Pacific Biosciences) (5) to filter the subreads. The genome sequence was 6,647,893 bp, and the G+C content was 67.0%. The assembly generated 4 contigs with an *N*_50_ contig size of 3,558,923 bp. Genome annotation was performed using CRITICA (6) and Glimmer2 (7), and 9,329 predicted coding sequences were identified. In addition, 65 tRNA genes and 15 rRNA genes were identified using tRNAscan-SE (8) and BLASTN (9), respectively.

### Nucleotide sequence accession numbers.

The nucleotide sequence and annotation data for the *Burkholderia* sp. CCA53 draft genome have been deposited in DDBJ/EMBL/GenBank under accession numbers BDDJ01000001 to BDDJ01000004.
